# Spatial working memory in Alzheimer’s disease: A study using the Corsi block-tapping test

**DOI:** 10.1590/S1980-57642008DN10400011

**Published:** 2007

**Authors:** Carla Cristina Guariglia

**Affiliations:** MD, Behavioral and Cognitive Neurology Unit. Hospital das Clínicas of the University of São Paulo School of Medicine, São Paulo SP, Brazil.

**Keywords:** working memory, Alzheimer’s disease, Corsi block-tapping test, spatial memory, non-verbal memory, memória operacional, doença de Alzheimer, teste dos blocos de Corsi, memória espacial, memória não-verbal

## Abstract

The Corsi block-tapping test was developed as a non-verbal task to measure spatial memory. In this test, cubes are tapped by the examiner in novel sequences of increasing length after which participants are required to reproduce each sequence immediately.

**Objectives:**

To evaluate spatial working memory in Alzheimer’s disease (AD) patients.

**Methods:**

30 elderly control subjects (21 women, 9 men) and 30 patients with probable Alzheimer’s disease (15 women and 15 men), with 8 or more years of schooling, were evaluated with the Mini-Mental State Examination (MMSE), digit span and Corsi block-tapping test. Proportions were compared using Chi-Square, and continuous variables with the Mann-Whitney tests.

**Results:**

AD patients were older than controls (p=0.014), but there were no differences regarding gender or educational level between these groups. The performance on the Corsi block-tapping test differed between AD and control individuals (p=0.010), and between patients with moderate dementia and controls (p=0.032), but not between control individuals and patients with mild dementia (p=0.090).

**Conclusions:**

In the present study, AD patients with moderate dementia showed impairment in spatial working memory while those with mild dementia did not. This finding may be due to the relatively small sample size, but it is also possible that spatial memory may be normal in the initial (limbic) phase of AD.

## Abstract (pt)

O teste dos blocos de Corsi (TBC) foi desenvolvido como uma medida não verbal da memória operacional espacial (MOE). Os blocos devem ser tocados pelo examinador em seqüências crescentes e os participantes devem repetir cada seqüência.

**Objetivos:**

Avaliar a MOE em pacientes com DA.

**Métodos:**

30 sujeitos controles (21 mulheres, 9 homens) e 30 pacientes com DA provável (15 mulheres, 15 homens) com escolaridade superior a 8 anos foram avaliados através do Mini Exame do Estado Mental (MEEM), extensão de dígitos, fluência verbal e TBC. Os dados foram comparados através do teste do qui-quadrado e as variáveis contínuas através do teste de Mann-Whitney.

**Resultados:**

Pacientes com DA eram mais velhos (p=0,014), mas não houve diferença quanto ao gênero ou escolaridade. O desempenho no teste dos blocos de Corsi foi diferente entre os pacientes com DA e sujeitos controles (p=0,010) e entre pacientes com demência de intensidade moderada e controles (p=0,032), mas não houve diferença entre pacientes com DA leve e controles (p=0,090).

**Conclusões:**

No presente estudo, pacientes com DA moderada demonstraram comprometimento na memória operacional espacial, o que não foi demonstrado nos pacientes com demência leve. Este achado pode estar relacionado ao pequeno tamanho da amostra, mas também é possível que a memória operacional espacial esteja normal na fase inicial (límbica) da DA.

In 1974, Baddeley and Hitch proposed a three-component model of working memory to which, after a few years, the addition of a further component was proposed.^1^ The current model of working memory comprises the central executive, phonological loop, visuospatial sketch pad and episodic buffer. The visuospatial sketch pad is the subsidiary system of working memory responsible for visuospatial working memory. It integrates spatial and visual information into a representation which may be temporarily stored and manipulated. The system is involved for example in reading, an action that requires maintaining a representation of the page and its layout that will remain stable to facilitate eye movements from the end of one line to the beginning of the next.^1,2^

Ungergleider et al. (1998) evaluated normal subjects with PET scans in a task where subjects had to memorize faces and their locations. It was found that different types of working memory activated separate prefrontal regions. Working memory for objects activates predominantly inferior prefrontal cortex whereas working memory for spatial locations activates the superior prefrontal cortex.^3^

Functional magnetic resonance imaging (fMRI) studies have shown that in addition to the dorsolateral prefrontal cortex, the posterior parietal cortex also plays a role in spatial working memory.^4^

The Corsi block-tapping test was developed in 1971 as a measure of spatial memory with minimal verbal mediation.^5^

The test requires the maintenance of a visuospatial pattern and a movement sequence. Retaining a sequence of targeted movements requires memory both for observed movement and for a pathway between objects.^2^

In the same way the digit span is influenced by educational level,^6^ the Corsi block- tapping test is sensitive to age, gender and schooling years, only there is no hemispheric lateralization for this test.^2,4^

Akin to the orally presented digit span sequences, the number of blocks is a determinant of difficulty. Thus, the level of difficulty increases with the number of blocks in a sequence while performance generally declines with increased span load.^7^

Patients with Alzheimer’s disease (AD) show decline in immediate memory span and in working memory as the disease progresses because of a deficit in the central executive component of working memory. This hypothesis is supported by Morris and Baddeley who compared AD patients and normal elderly in a combined simultaneous task.^8^

Previous studies which have evaluated spatial working memory in AD patients found that this group had impairment of immediate visuospatial memory.^9-11^

The aim of the present study was to analyze spatial working memory in Alzheimer’s disease patients through the Corsi block-tapping test, and to compare the performances of patients with mild and moderate dementia.

## Methods

We evaluated 30 subjects and 30 patients with probable AD from the Cognitive and Behavioral Neurology Unit of the Hospital das Clínicas of the University of São Paulo School of Medicine. All data were collected after analysis and approval by the ethics Committee of the institution. Informed consent was obtained from all participants.

AD patients were diagnosed following the NINCDS-ADRDA criteria^12^ and control subjects were submitted to a structured memory questionnaire to exclude cognitive impaired subjects. Both groups had at least 8 years of schooling. Patients with depression or other neurological disease were excluded.

The evaluation consisted of a brief cognitive battery which included the Mini-Mental State Examination (MMSE), digit span and verbal fluency tests.^13-16^

Both groups were also evaluated with the Corsi block-tapping test. The original apparatus for the task consisted of a set of nine 3.1 cm cubes arranged irregularly on a board with a numbered side that only the examiner can see. The examiner taps the cubes in a sequence and the participant is required to reproduce the sequence in the same order immediately after demonstration. Subsequently, the examiner taps another sequence of increased length, followed by the patient and so forth until a maximum sequence of nine cubes is reached.^5^ The result was scored by the number in the maximum sequence that the patient was able to reproduce correctly, giving a maximum possible score of nine.

After the initial evaluation, patients were classified into mild or moderate dementia according to their scores on the MMSE, 19 points or more for mild dementia and between 14 and 18 for moderate dementia.

### Statistical analysis

All variables were analyzed from a descriptive viewpoint, with determination of means and standard deviations. The chi-square test was used to verify the difference among categorical variables and the Mann-Whitney test for quantitative variables. The value of statistical significance accepted was 0.05. The Statistical Package for the Social Sciences for Windows, version 10.0 (SPSS Inc) was used.

## Results

The results of demographic data are presented in Table 1 while Table 2 shows the mean scores on the cognitive tests, including the Corsi block-tapping test.

**Table 1:** Demographic data. Mean and standard deviation (SD).

	Control subjects N=30	Patients N=30	p	Mild dementia patients N=15	p1	Moderate dementia patients N=15	p2
Gender	21 W; 9 M	15 W; 15 M	0.940	6 W; 9 M	0.128	7 W; 6 M	0.715
Ages (Mean, SD)	69.6±7.1	74.7±8.4	0.014	76.2±9.1	0.016	73.3±8.2	0.328
Years of schooling (Mean, SD)	11.0±2.6	11.0±3.1	0.747	10.8±3.7	0.428	11.3±2.6	0.267

M: men; W: women; p: comparison between patients and control subjects; p1: comparison between mild dementia patients and control subjects; p2: comparison between mild dementia patients and moderate dementia patients.

**Table 2:** Mean scores (and standard deviation) in the Mini-Mental State Examination, verbal fluency, digit span and Corsi Block-tapping test in control subjects and Alzheimer's disease patients, with mild or moderate dementia.

	Control subjects N=30	Patients N=30	p	Mild dementia patients N=15	Moderate dementia patients N=15	p1	p2	p3
MMSE	28.7±1.2	20.2±3.5	<0.001	23.06±1.9	17.26±1.5	0.000	0.000	0.000
Verbal fluency	17.6±5.8	11.0±4.3	0.004	10.6±3.5	9.2±3.6	0.000	0.000	0.436
Digit span	6.2±0.9	4.5±1.9	0.009	3.4±2.5	2.2±1.8	0.001	0.000	0.174
Corsi block test	4.8±1.0	3.9±1.3	0.010	4.2±0.8	3.6±1.7	0.090	0.032	0.533

p: control subjects compared with all patients; p1: control subjects compared with mild dementia patients; p2: control subjects compared with moderate dementia patients; p3: mild dementia patients compared with moderate dementia patients.

AD patients had a worse performance in all tests, including the Corsi block-tapping test, versus controls. There were significant differences between mild AD patients and control subjects in the Corsi block-tapping test, but the test was unable to differentiate between mild and moderate AD patients.

## Discussion

In this study, AD patients exhibited a significantly poorer performance on the Corsi block-tapping test, which suggests impairment in visuospatial working memory. Impairment was only seen in AD patients with moderate dementia.

Other studies have previously tested AD patients using the Corsi block-tapping test.^9-11^

Liu et al. (1991) studied 15 AD patients with mean MMSE scores of 22.87, along with 15 controls, and found that AD patients were impaired in the Corsi block-tapping test.^9^ Grossi et al. (1993) evaluated 39 patients with AD using the Corsi block-tapping test and found that patients with mild dementia also showed impairment in working memory,^10^ while Trojano et al. (1994) compared two different versions of the Corsi block-tapping test in AD patients and concluded that subjects were impaired in both versions.^11^

All of the above-mentioned studies, as did the present study, have shown that the AD patients were impaired in visuospatial working memory upon testing by the Corsi’s block tapping test.

Liu’s MMSE results were similar to those seen in our mild dementia group. However, their patients with mild AD already demonstrated impaired performance on the Corsi block-tapping test, a result not seen in our mild dementia group. In the present study, no confirmation that AD patients with mild dementia had impaired spatial working memory was evident. This finding may be related to the relatively small sample size, but it is also possible that spatial memory may be normal in the initial (limbic) phase of AD.

Since it is known that working memory declines with age, especially in the visuospatial domain,^17^ together with the fact that our patients with mild dementia were older than controls, the absence of any performance difference between controls and mild dementia subjects on the Corsi block-tapping test represented a somewhat unexpected finding.

## References

[1] Baddeley A (2003). Working memory and language: an overview. J Commun Disord.

[2] Della Sala S, Logie RH, Baddeley Alan D., Kopelman Michael D., Wilson Barbara A. (2002). Neuropsychological impairments of visuals and spatial working
memory. The handbook of memory disorders.

[3] Ungerleider LG, Courtney SF, Haxby JB (1998). A neural system for human visual working memory. Proc Natl Acad Sci.

[4] Van Asselen M, Kessels RPC, Neggers SFW, Kapelle LJ, Frijns CJM, Postma A (2006). Brain areas involved in spatial working memory. Neuropsychologia.

[5] Milner B (1971). Interhemispheric differences in the localization of psychological
processes in man. Br Med Bull.

[6] Souza-Talarico JN, Caramelli P, Nitrini R, Chaves EC (2007). The influence of schooling on working memory performance in
elderly individuals without cognitive decline. Dement Neuropsychol.

[7] Busch RM, Farrel K, Lisdahl-Medina K, Orian RK (2005). Corsi block-tapping task performance as a function of path
configuration. J Clin Exp Neuropsychol.

[8] Baddeley AD, Bressi S, Della Sala S, Logie R, Spinnler H (1991). The decline of working memory in Alzheimer's
disease. Brain.

[9] Liu L, Gauthier L, Gauthier S (1991). Spatial disorientation in persons with early senile dementia of
the Alzheimer's Type. Am J Occup Ther.

[10] Grossi D, Becker JT, Smith C, Trojano L (1993). Memory for visuospatial patterns in Alzheimer's
disease. Psychol Med.

[11] Trojano L, Chiacchio L, Luca G, Fragassi NA, Grossi D (1994). Effect of testing procedure on Corsi's block tapping test in
normal subjects and Alzheimer's disease. Percept Mot Skills.

[12] McKhan G, Drachman D, Folstein M, Katzman R, Price D, Stadlan EM (1984). Clinical diagnosis of Alzheimer's Disease:report of the
NINCDS-ADRDA Work Group under the auspices of Department of Health and Human
Services Task Force on Alzheimer´s Disease. Neurology.

[13] Nitrini R, Caramelli P, Herrera Junior E (2004). Performance of illiterate and literate nondemented elderly
subjects in two tests of long-term memory. J Int Neuropsychol Soc.

[14] Nitrini R, Lefevre BH, Mathias SC (1994). Neuropsychological tests of simple application for diagnosing
dementia. Arq Neuropsiquiatr.

[15] Folstein MF, Folstein SE, McHugh PR (1975). Mini-Mental State: a practical method for grading the cognitive
state of patients for the clinician. J Psychiatr Res.

[16] Brucki SM, Nitrini R, Caramelli P, Bertolucci PH, Okamoto IH (2003). Suggestions for utilization of the mini-mental state examination
in Brazil. Arq Neuropsiquiatr.

[17] Hale S, Myerson J, Emery L J, Lawrence B M, DuFault C, Conway ARA, Jarrold C, Kane M J, Miyake A, Towse JN (2007). Variation in working memory across the life span. Variation in Working Memory.

